# Hemophilia A subjects with an intron-22 gene inversion mutation show CD4^+^ T-effector responses to multiple epitopes in FVIII

**DOI:** 10.3389/fimmu.2023.1128641

**Published:** 2023-03-01

**Authors:** Devi Gunasekera, Pooja Vir, Ahmad Faisal Karim, Margaret V. Ragni, Kathleen P. Pratt

**Affiliations:** ^1^ Department of Medicine, Uniformed Services University of the Health Sciences, Bethesda, MD, United States; ^2^ Henry Jackson Foundation for the Advancement of Military Medicine, Bethesda, MD, United States; ^3^ Department of Medicine, University of Pittsburgh, Pittsburgh, PA, United States

**Keywords:** hemophilia A, immune tolerance, intron-22 inversion mutation, epitope mapping, factor VIII

## Abstract

**Background:**

Almost half of severe hemophilia A (HA) is caused by an intron 22 inversion mutation (Int22Inv), which disrupts the 26-exon *F8* gene. Inverted *F8* mRNA exons 1-22 are transcribed, while *F8B* mRNA, containing *F8* exons 23-26, is transcribed from a promoter within intron 22. Neither FVIII activity nor FVIII antigen (cross-reacting material, CRM) are detectable in plasma of patients with an intron-22 inversion.

**Objectives:**

To test the hypothesis that (putative) intracellular synthesis of FVIII proteins encoded by inverted *F8* and *F8B* mRNAs confers T-cell tolerance to almost the entire FVIII sequence, and to evaluate the immunogenicity of the region encoded by the *F8* exon 22-23 junction sequence.

**Patients/Methods:**

Peripheral blood mononuclear cells (PBMCs) from 30 severe or moderate HA subjects (17 with an Int22Inv mutation) were tested by ELISPOT assays to detect cytokine secretion in response to FVIII proteins and peptides and to map immunodominant T-cell epitopes. Potential immunogenicity of FVIII sequences encoded by the *F8* exon 22-23 junction region was also tested using peptide-MHCII binding assays.

**Results:**

Eight of the Int22Inv subjects showed robust cytokine secretion from PBMCs stimulated with FVIII proteins and/or peptides, consistent with earlier publications from the Conti-Fine group. Peptide ELISPOT assays identified immunogenic regions of FVIII. Specificity for sequences encoded within *F8* mRNA exons 1-22 and *F8B* mRNA was confirmed by staining Int22Inv CD4^+^ T cells with peptide-loaded HLA-Class II tetramers. FVIII peptides spanning the *F8* exon 22-23 junction (encoding M2124-V2125) showed limited binding to MHCII proteins and low immunogenicity, with cytokine secretion from only one Int22Inv subject.

**Conclusions:**

PBMCs from multiple subjects with an Int22Inv mutation, with and without a current FVIII inhibitor, responded to FVIII epitopes. Furthermore, the FVIII region encoded by the exon 22-23 junction sequence was not remarkably immunoreactive and is therefore unlikely to contain an immunodominant, promiscuous CD4^+^ T-cell epitope. Our results indicate that putative intracellular expression of partial FVIII proteins does not confer T-cell tolerance to FVIII regions encoded by inverted *F8* mRNA or *F8B* mRNA.

## Introduction

Almost half of severe hemophilia A (HA) patients have a *F8* intron-22 inversion mutation (Int22Inv), which precludes expression of an intact, functional FVIII protein (1, 2). The *F8* gene consists of 26 exons encoding FVIII domains A1-A2-B-A3-C1-C2 (3). An additional transcription start site within intron 22 produces *F8B* mRNA, which consists of a short exon encoding eight non-FVIII amino acid residues followed by *F8* exons 23-26 encoding V2125-Y2332 (4). *F8B* mRNA is expressed in multiple tissues of individuals with and without HA, with the exception of HA patients having a deletion mutation within exons 23-26. In addition to *F8B* mRNA, individuals with an intron-22 inversion mutation express a truncated *F8* mRNA consisting of *F8* exons 1-22 (1, 2).

Approximately 30% of severe HA patients develop neutralizing anti-FVIII antibodies following replacement therapy with exogenous FVIII, which are clinically referred to as “inhibitors” (5–7) due to their interference with normal blood coagulation *via* blocking of FVIII cofactor activity. The development of anti-FVIII antibodies in HA patients follows a classic prime-boost response, with inhibitors generally developing within the initial 20 exposures to therapeutic FVIII (8). Initial inhibitor development after 150+ FVIII infusions is far less common. The role of CD4^+^ T cells in providing ongoing help necessary for anti-FVIII antibody generation was clearly shown in a 1993 study of HA patients infected with HIV. For those with an inhibitor, their titers declined as their CD4^+^ T-cell counts decreased (9). Proliferation and cytokine secretion of CD4^+^ T cells from HA subjects in response to FVIII protein and synthetic peptides was demonstrated in subsequent studies (10–15). More recently, specific HLA-restricted T-cell epitopes have been identified, with several confirmed by isolation of T-cell clones responding to specific FVIII peptides (16–21). Thus, the essential role of CD4^+^ T cells in development and persistence of inhibitor responses is well established.

**Figure 1:**
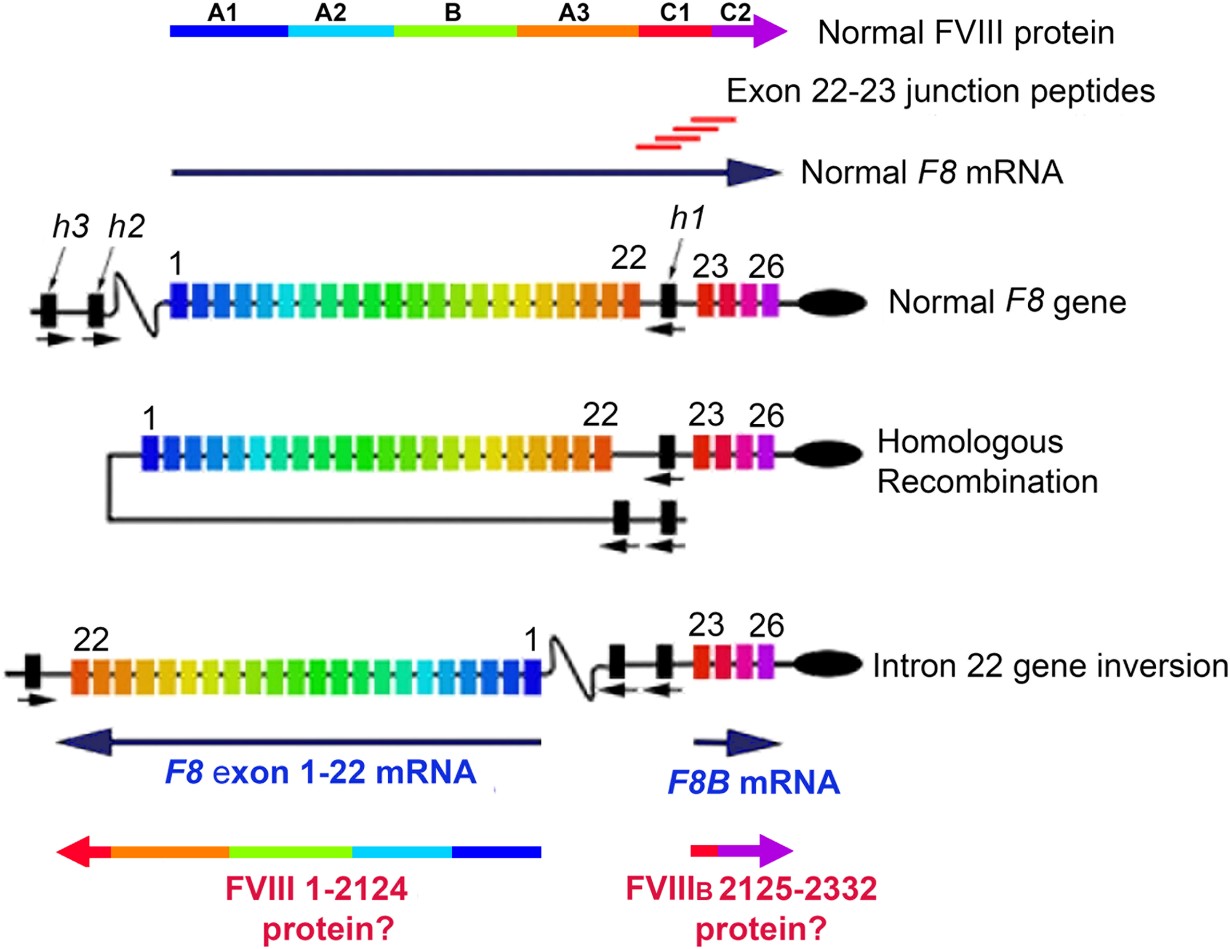
FVIII Structure, Int22Inv DNA, Int22Inv mRNA, and hypothesized partial FVIII proteins. Full-length FVIII consists of domains A1, A2, A3, B, C1 and C2, which are encoded by the 26-exon *F8* gene. Following a type 1 or type 2 intron 22 inversion mutation, the primary *F8* mRNA product consists of *F8* exons 1-22, which encode the FVIII A1, A2 and B domains, plus the C1 domain sequence through M2124 and then terminating at a stop codon within intron 22. The shorter transcript *F8B* is comprised of 24 nucleotides followed by *F8* exons 23-26, which encode FVIII residues 2125-2332. If these two partial FVIII proteins were expressed intracellularly, this could in principle promote tolerance to FVIII as a “self”-protein. The 20-mer exon 22-23 junction peptides, with *F8* sequences overlapping by 12 residues, were designed to allow peptides to bind to the HLA-DRB1 binding grooves in multiple possible registers, in order to evaluate their potential ability to be presented to CD4^+^ T cells.

A 2009 case-control study indicated that large *F8* gene deletions were associated with increased risk of developing an inhibitor (22), and a 2012 meta-analysis further indicated that inhibitor risk in HA-Int22Inv patients was somewhat lower than in severe HA patients with a large structural alteration of the *F8* gene, e.g., large deletion or early nonsense mutations (23). This provided some support for an earlier hypothesis that endogenous intracellular expression of FVIII partial proteins from these two mRNAs occurs, and that it may confer self-tolerance to the corresponding FVIII protein sequences, thereby decreasing the immunogenicity of FVIII replacement therapy in Int22Inv patients (24, 25). Since a substantial fraction of Int22Inv patients still develop inhibitors, a corollary of this hypothesis is that the FVIII C1 domain region encoded by mRNA spanning the exon 22-23 splice site (corresponding to FVIII residues ~I2103-A2146), which would be the only “non-self” region of therapeutic FVIII (**Figure 1**), is a promiscuous, highly immunogenic neo-epitope.

The present study tests this hypothesis and corollary by directly querying CD4^+^ T-effector responses of HA subjects with and without an Int22Inv mutation to recombinant (r)FVIII proteins and synthetic peptides. A primary goal was to map immunodominant epitopes that elicit CD4^+^ T-cell responses in the Int22Inv subjects and determine if the results indicate tolerance to FVIII regions encoded by inverted *F8* exons 1-22 and/or by *F8B*. In addition, peptide-HLA Class II binding assays were employed to test the ability of peptides encoded by the *F8* exon 22-23 junction region to be presented on a representative series of recombinant HLA-DRB1 proteins. The latter experiments would indicate if peptides encoded by this junction region, which cannot be expressed in patients with an Int22Inv mutation, could represent a promiscuous, immunodominant epitope responsible for inhibitor development in Int22Inv patients.

## Materials and methods

### Human subjects and blood samples

Blood samples from 11 severe and moderately severe HA subjects were donated as part of the “INHIBIT” feasibility study (5U34HL 114674, MVR, PI). Three of these were known Int22Inv subjects, while the HA-causing mutation was unknown for six of these subjects. PBMCs from severe HA and normal control subjects that were banked from earlier studies were also utilized (USU IRB#1, protocols MED-83-3442 and MED-83-3426). All subjects provided informed consent consistent with the Principles of Helsinki. Additional de-identified normal control samples were obtained from the NIH Blood Bank (Exempt E4). Both freshly prepared and frozen (7% DMSO in FBS) PBMC samples were analyzed.

### Inhibitor titers

Inhibitor titers, expressed as Bethesda units (BU)/mL, were from clinical records for the subjects, which reported results of CLIA-certified clotting or chromogenic substrate assays (26). Both clotting and chromogenic inhibitor assays are functional assays that measure the inhibition of FVIII cofactor activity in the presence of anti-FVIII antibodies. The clotting assay measures plasma clotting time [using a 1-stage assay (27)], while the chromogenic assay measures cleavage of a chromogenic substrate for factor X; normal factor X kinetics require a normal FVIII level. In either assay, serial dilutions of the patient plasma are mixed with a normal pooled plasma sample, and the residual FVIII activity, compared to a parallel assay using the normal pooled plasma plus buffer, is measured and reported in BU/mL. One BU/mL is the reciprocal of the patient plasma dilution that results in 50% normal FVIII activity in the assay. The lower limit of quantification is generally considered to be 0.6 BU/mL, and titers > 5.0 BU/mL are considered high-titer inhibitors.

### Reagents, buffers and instruments

Recombinant (r)FVIII (Kogenate 27NZPO) was from Bayer (Leverkusen, Germany) or Baxter (Westlake Village, CA, USA; Recombinate TRH07504AA). Recombinant FVIII-C2 protein was purified in-house as described (28). Tetanus and Diptheria (TD) toxoid adsorbed was from Mass Biologics, (Boston, MA, USA) and Remel PHA (R30852801) (100μg/ml) were from Thermo Scientific (Rockford, IL, USA). Benzonase nuclease, 250 U/μl (021M0852) and 0.4% Trypan blue (T8154) were from Sigma Aldrich (St Louis, MO, USA). RPMI 1640 with 25 mM HEPES, DMEM/F-12(1(1:1), MEM nonessential amino acids (100X), 1X D-PBS (Ca/Mg-free) and 10X D-PBS were from Life Technologies (Grand Island, NY, USA). Ficoll-Paque PLUS was from GE Healthcare Life Sciences (Piscataway, NJ, USA). HCl was from JT Baker Tyco Products (Phillipsburg, NJ, USA). Human IFN-gamma ELISPOT pair (551849), Human IL-5 ELISPOT pair (551886), Human IL-10 ELISPOT pair (551883), AEC substrate set (551951) and TMB substrate were from BD Biosciences (San Jose, CA, USA). IL-7 (0.5ng/ml) was from Peprotech (Rocky Hill, NJ, USA). Fetal bovine serum was from Hyclone (Logan, UT, USA). ImmunO human serum type AB was from MP Biomedicals (Solon, OH, USA). Recombinant, extracellular domains of 10 HLA-DRB1 protein monomers, HLA Class II tetramers loaded with the relevant peptides and anti-HLA-DR antibody L243 were provided by the Tetramer Core Facility at the Benaroya Research Institute (Seattle, WA, USA). AIM-V medium and goat anti human IgG-HRP were from Invitrogen (Carlsbad, CA, USA). Sheep anti-human FVIII was from Cedarlane labs (Burlington, NC, USA). ELISA 12 well strips were from E & K Scientific (Santa Clara, CA, USA). Wallac enhancement solution, fluorescence assay buffer and europium-labeled straptavidin were from Wallac-Perkin Elmer (Turku, Finland). 96-well polypropylene plates and Corning Costar high-binding ELISA plates were from Sigma Aldrich (St. Louis, MO, USA), cat # CLS3799 and CLS2592, respectively). Recombinant human IL-2 (Chiron Il-2, Proleukin, 70 IU/ml) was from R & D systems, cat #202-IL-010)

### FVIII peptides and peptide pools

Individual 20-mer peptides spanning the FVIII A2, C1 and C2 sequences, as well as equimolar pools containing 2-5 of these peptides, were from Anaspec, Inc. (Fremont, CA, USA, **Table S1**). Additional 15-mer peptides spanning the FVIII A1, A2, A3, C1 and C2 domain sequences, with 12-residue overlaps, were from Mimotopes, Inc. (Mulgrave, Victoria, Australia), which also provided large, medium and small equimolar pools of these peptides (**Tables S2–S5**). An intron 22-23 ‘junction peptide pool’ was created by combining four 20-mer peptides (Mimotopes, Inc.) with overlapping sequences spanning the *F8* exon 22-23 – encoded region (FVIII 2103-2146) in DMSO (**Table 1**). All peptides were ordered at >70% advertised purity and their quality was verified by mass spectrometry.

**Table 1:** FVIII peptides spanning the F8 exon 22-23 junction region.

2103-2122	IMYSLDGKKWQTYRGNSTGT
2111-2130	KWQTYRGNSTGTL**MV**FFGNV
2119-2138	STGTL**MV**FFGNVDSSGIKHN
2127-2146	FGNVDSSGIKHNIGNPPIIA

The bolded M and V indicate residues M2124 and V2125, which are the last amino acid encoded by exon 22 and the first amino acid encoded by exon 23, respectively.

### Peptide-MHCII binding assays and predictions

Quantitative peptide-MHC competition binding assays were carried out to determine binding avidities of four 20-mer peptides spanning the *F8* exon 22-23 junction region to 10 HLA-DRB1 proteins using methodology described previously (29). Briefly, 1-µl aliquots of serially diluted (50nM – 50μM in DMSO) non-biotinylated FVIII peptides, were added to triplicate wells of 96-well polypropylene plates. Serially diluted non-FVIII reference peptides known to bind to specific HLA-DRB1 (**Table S6**) were added to separate wells as positive controls. The HLA-DRB1 proteins were diluted to 50 nM in 150 mM citrate-phosphate, pH 5.4, 0.75% n-octyl-beta-D-glucopyranoside, 1 mM PMSF. 50 μl of the HLA-DRB1 solution was added per well, and the plates were sealed and incubated at 37°C for 30 min to allow peptide binding to the HLA-DRB1 protein. Next, 1 μl of the appropriate biotinylated reference peptide, at a concentration in the known binding range for its respective HLA-DRB1 (**Table S6**), was added to each well, the solutions were mixed, and the plates were incubated at 37°C for 18-24 hrs. 100 μl aliquots of the anti-HLA-DR antibody L243 (10 μg/mL in 12.5 mM borate buffer, pH 8.2) were added to 96-well ELISA plates, and the plates were incubated at 4°C for 18-24 hrs, washed, and blocked with 1xDPBS containing 5% FBS for 3 hrs. The plates were washed again and 50 μl neutralization solution (50 mM Tris, pH 8.0, 0.75% n-octyl-beta-D-glucopyranoside) was added to each well. The contents of wells containing the peptide-HLA-DRB1 binding reactions were then transferred to wells of the ELISA plates containing the neutralization solution and the ELISA plates were incubated for 18-24 hrs at 4°C. After washing the plates 5X with 300 ul/well of 1XPBS, 0.05% Tween-20, 100 ul of Europium-labeled streptavidin (diluted 1:1,000 into Wallac assay buffer) was added to each well. The plates were covered with a black polystyrene lid, incubated at room temperature for 4 hrs and washed 5X with 300 ul/well of 1XPBS, 0.05% Tween-20. Wallac enhancement solution (100 μl/well) was then added, and the plates were incubated at room temperature for 30 min and then read on a Victor 2D time-resolved fluorometer. Sigmoidal binding curves were simulated and IC50 values calculated for the FVIII peptides, based on their competition with the reference peptides for each HLA-DRB1.

Predicted affinities of the four FVIII junction peptides for the 10 HLA-DRB1 alleles were obtained using the Immune Epitope Database (IEDB) server, using the NetMHCIIPan 4.0 prediction algorithm (30).

### *In vitro* expansion of FVIII-specific T cells

FVIII-specific CD4^+^ T cells were expanded by culturing PBMCs with or without rFVIII, rFVIII-C2 protein, or pooled or individual FVIII peptides, as follows. One vial of frozen PBMCs (~10 million cells) was thawed by slowly adding 9 ml of AIM-V medium containing 10% human serum + 1.8 μl of 25K benzonase nuclease. The cells were washed with AIM-V medium containing 10% human serum and re-suspended in AIM-V medium containing 15% human serum, and then plated at 1x10^6^ cells per well in 48-well cell culture plates (1 mL/well) with one of the following: rFVIII protein (1 μg/mL ~8nM), rFVIII-C2 protein (2 μg/mL = 50 nM), pooled FVIII peptides (50 nM and 70 nM), FVIII pooled junction peptides (200 nM), or individual FVIII peptides (50 or 100 nM). Positive control wells were stimulated with 0.02Lf (5 μl/mL) TD toxoid, PHA (10 μg/mL) and negative controls with 5 μl DMSO. The plates were incubated for five days at 37°C in a 5% CO_2_ incubator. At day 6, cells were harvested and re-suspended in AIM-V medium (no serum) containing 0.5ng/mL IL-7 and then directly plated on 96-well flat-bottom ELISPOT plates. For some individual peptide ELISPOT assays, expansions were continued for an additional 3 days, adding fresh AIM-V medium and IL-2 (3.5 IU/mL final concentration).

### Epitope mapping by ELISPOT assays

ELISPOT plates were coated with capture antibody solutions (anti-IFN-γ, anti-IL-5 or anti-IL-10) diluted to 5 μg/ml in DPBS and incubated at 4C overnight. Wells were then washed once with 200 μl/well Blocking Solution (AIM-V medium containing 10% human serum). Another 200 μl Blocking Solution was then added to each well and the plates were incubated for 2 hrs at room temp and the Blocking solution discarded. The expanded PBMCs were then added in serial dilutions (carried out in duplicate when sufficient cells were available) with final concentrations of 2X10^5^, 1x10^5^ and 5x10^4^ cells/well, and incubated with the same antigenic stimulants as before. The plates were incubated for 24 hrs for IFN-γ ELISPOTS or 48 hrs for IL-5 and IL-10 ELISPOTS at 37C, 5% CO_2_. The suspended cells were then discarded and the plates washed with 200 μl/well dH_2_O (2X) and 3x with PBS containing 0.05% Tween 20 (wash buffer I). The biotinylated anti-human antibody provided in the kits (0.25 μg/ml, diluted in PBS + 10% human serum) was then added and the plates were incubated for 2 hrs at room temperature. The plates were washed 3X with 200 μl/well wash buffer I, washed 2X with 200 μl/well PBS, and developed with streptavidin-HRP (BD Biosciences, #557630) diluted 1:100 in PBS containing 10% human serum for 1 hr at room temperature. Plates were then washed 4X with 200 μl/well wash buffer I, and washed 2X with 200 μl/well PBS. Spots were then developed by adding 100 μl/well of BD ELISPOT AEC substrate and incubated for 5-30 minutes in the dark. The wells were then washed 2X with 200 μl/well dH_2_0 and air-dried at room temp for 2 hrs. IFN-γ, IL-5 and IL-10-specific spot-forming cells (SFC) were then counted using software on a CTL Immunospot S6 Ultimate Image Analyzer. Stimulants were: rFVIII 5 μl/mL = 5 nM; rFVIII-C2 1μg/mL = 50 nM; FVIII peptides (pooled or individual): 50-100 nM; TT; 5 μl/mL; PHA: 5 μl/mL. The criteria for antigen-specific (positive) responses were: a minimum of 25 spots per million cells (based on spots per 200,000 cells/well) and wells with the stimulant had ≥3X the average number of spots counted for the unstimulated wells. In addition, antigen-specific responses to large FVIII peptide pools were “decoded”, when sufficient cells were available, by subsequent ELISPOT assays using smaller peptide pools and then with individual peptides, to map immunogenic FVIII regions and specific epitopes contributing to the cytokine response.

### Epitope mapping by HLA Class II tetramer staining

PBMCs were added to 48-well plates in 1ml of 10% human serum RPMI medium (1 million PBMCs per well) and then stimulated with individual or pooled 20-mer or 15-mer FVIII peptides (10 μM) and incubated for up to 19 days at 37C, 5% CO_2_. At day 7, 50 μl of IL-2 was added (35 IU/mL final concentration) without removing any medium. At day 9, 400-500 μL supernatant was removed and replaced with fresh 15% human serum RPMI medium containing 50 μl IL-2 after which cells were fed with 50 μl IL-2 every 48-72 hours, splitting as needed. After 17-19 days, the cells were re-suspended, 100 μL aliquots were transferred to FACS tubes, and 1.5 μl of the appropriate peptide-loaded, PE-labeled HLA Class II tetramer (~10 μg/mL final concentration) was added. The tubes were incubated in the dark at 37C for 2 hrs and then put on ice. A mixture of anti-CD3-PerCP + anti-CD4 APC (3.75 μL each from stock solutions) was added to each tube, and tubes with no antibody added served as a negative staining control. The tubes were then incubated in the dark at 4C for 30 min. Additional tubes containing beads for compensation (ThermoFisher (ebiosciences), Rockville, MD, USA), were stained by adding 5 μL of anti-CD3-PerCP, 1.5 μl of anti-CD4-APC and 1.5 μl of anti-CD4-PE and tubes were incubated in the dark for 30 min. Sample and control cells were then washed and re-suspended in ice-cold FACS buffer, stored on ice in a covered container, and analyzed by flow cytometry and/or FACS acquisition. The gating strategy was: Singlets (FSC-H/FSC-A), Lymphocytes (SSC-A/FSC-A), followed by gating on anti-CD3-PerCP-high, followed by tetramer-PE/CD4-APC to detect antigen-specific CD4+ T cells.

### Isolation of FVIII-specific T-cell clones

To isolate FVIII-specific T-cell clones, CD4^hi^Tetramer^hi^ cells were single-cell sorted into 96-well round-bottom plates containing 100 μl RPMI medium per well. To each well, (200,000) irradiated HLA-mismatched PBMC were added as feeder cells in a volume of 100 μL RPMI Medium containing 15% human serum AB plus PHA (5 μg/mL). The cells were incubated for 24 hrs at 37C, 5% CO_2_, and then IL-2 (10 IU/mL) in 25 μl RPMI medium was added to each well. The cells were then incubated at 37C in 5% CO_2_ for another 14-21 days. For wells containing expanding clones, tetramer staining was performed to confirm their specificity. If clones were not expanding, the cells were re-stimulated with irradiated, HLA-mismatched PBMCs and PHA as before, adding fresh medium and IL-2 (3.5 IU/mL final concentration) as needed to continue the expansion or else frozen in 10% DMSO in FBS.

## Results

### Peptide-MHCII binding assays and predictions

The experimental FVIII peptide-HLA-DRB1 affinities are in **Table 2A** and their predicted affinities are in **Table 2B**. Only HLA-DR0404, DR0701, DR0901 and DR1501 showed moderate or strong binding affinity to one or more peptides spanning the *F8* exon 22-23-encoded region of FVIII, while weak binding was observed to HLA-DR0101 and DR0401. The MHCIIpan algorithm (30) predicted moderate-to-strong binding affinity to additional HLA-DR proteins. Based on the experimental results, ELISPOT assays were then carried out using PBMCs from nine HA subjects with an intron 22 inversion mutation who were also known to have *HLA-DR1, DR4, DR7, DR9* or *DR15* alleles.

**Table 2A:** Experimental binding affinities (IC50 values) of FVIII exon 22-23 junction peptides.

FVIII peptide	DR0101	DR0301	DR0401	DR0404	DR0701	DR0901	DR1001	DR1101	DR1104	DR1501
2103-2122	n.b.	61.2	238.07	n.b.	n.b.	57.3	n.b.	50.58	n.b.	41.95
2111-2130	10.91	227.6	43.66	127.5	5.9	0.6	352.9	68.83	n.b.	9.09
2119-2138	n.b.	n.b.	74.46	0.9	1853.08	55.6	n.b.	n.b.	n.b.	2.97
2127-2146	n.b.	n.b.	n.b.	43.8	n.b.	60.9	n.b.	n.b.	n.b.	7.52

0-1.0 μM (Strong binding, dark gray); >1.0 and <10.0 μM (Moderate binding, medium gray); 10.0-50.0 μM (Weak binding, light gray); >50 μM (no binding, n.b.).

**Table 2B:** Predicted binding affinities (IC50 values)* of FVIII exon 22-23 junction peptides.

FVIII peptide	DR0101	DR0301	DR0401	DR0404	DR0701	DR0901	DR1001	DR1101	DR1104	DR1501
2103-2122	0.415	2.074	2.045	2.172	1.315	0.974	0.768	1.082	1.063	0.356
2111-2130	0.189	6.081	0.515	1.816	0.256	0.323	0.318	1.764	2.908	0.857
2119-2138	0.188	3.948	0.164	0.186	0.161	0.26	0.145	1.897	1.934	0.209
2127-2146	0.45	4.058	1.053	2.044	0.983	1.384	1.113	2.195	2.799	1.452

* predictions run on the IEDB website (http://tools.iedb.org/mhcii) 11/27/22 and 1/16/23 using NetMHC Pan 4.0^15,16^.

0-1.0 μM (Strong binding, dark gray); >1.0 and <10.0 μM (Moderate binding, medium gray).

### Epitope mapping by ELISPOT assays

Several strategies were used to map T-cell epitopes, in order to test specific hypotheses and/or to rationally choose which peptides to utilize when there were not sufficient PBMCs to systematically test responses to larger and then smaller peptide pools followed by individual peptides (due to competing studies and resulting blood volume limitations in subjects with hemophilia A). The number of PBMCs per subject was highly variable, as samples were obtained from both adults and children, and the amounts of blood drawn also reflected the stated preferences of the subjects. Therefore, the number of experiments and replicate wells per experiment varied somewhat according to the number of cells available for the assays.

rFVIII and/or rFVIII-C2 domain protein ELISPOTs were included as a specific control in almost all ELISPOT experiments. For positive responders, IFN-γ secretion was generally more robust when cells were stimulated with 50 nM rFVIII-C2 than with 8 nM rFVIII. Unfortunately, FVIII protein becomes toxic to CD4^+^ T cells at higher concentrations. The stronger responses to rFVIII-C2, and to pooled or individual FVIII peptides in some assays, may simply be due to stimulation of lower-avidity CD4^+^ T cells by the higher concentrations of rFVIII-C2 and FVIII peptides. Of note, the rFVIII-C2 preps were routinely tested to confirm low endotoxin levels (31).

### Exon 22-23 junction peptides

Potential CD4^+^ T-cell responses to exon 22-23 junction sequences were tested using samples from nine HA subjects with an intron-22 inversion mutation who also had one of the *HLA-DRB1* alleles shown experimentally to bind peptides with these sequences: *HLA-DR1, DR4, DR7, DR9* or *DR15* (**Table 2A**). Eight of these subjects failed to show a response to the pooled FVIII exon 22-23 junction peptides when tested using IFN-γ ELISPOT assays (**Table 3**). Five of them had a current inhibitor and four had no inhibitor history. The one subject who showed a response to the junction peptides, G4, had a low-titer inhibitor. He had an intron 22 inversion mutation as well as a missense mutation, H1499Y, and his *HLA-DRB1* alleles were HLA-DRB1*0301, 1501. Therefore, additional MHCII binding predictions were made using the IEDB analysis resource Consensus tool (32, 33) with the input sequence FVIII 1485-1513, lpktsgkvellpkvhiyqkdlfptetsng, which contains all possible 15-mer sequences containing residue H1499. Interestingly, the IEDB prediction algorithm identified two overlapping 9-mer core sequences containing H1499 as potential HLA-DRB1*1501-restrictred CD4^+^ T-cell epitopes, ranking them in the top 15-20% based on predicted binding affinity (**Table S7**).

**Table 3:** IFN-γ ELiSpot responses of Int22Inv PBMCs to pooled junction peptides (FVIII 2103-2146).

Subject code	Age range	HLA-DRB1	Current inhibitor?	Past inhibitor that resolved?	Inhibitor peak (BU/mL)	F8 exon 22-23 junction peptide stimulation?
G1	18+	0701, 0701	N	N	0	N
G2	4-17	0901, 1101g	N	N	0	N
G3	18+	0701, 1301	Y	N	49	N
G4*	**18+**	**0302, 1501**	**Y**	**N**	**0.6**	**Y**
G5	4-17	0701, 1501	N	N	0	N
G6	18+	0801, 1503	Y	N	68	N
G7	4-17	0404, 1301	N	N	0	N
G8	4-17	0401, 0701	Y	N	1	N
G9	18+	0102, 1503	Y	N	>1000	N

*Subject G4 had an Int22Inv and a missense H1499Y mutation. His ELISPOT results were positive, showing 195 spots per million PBMCs, compared to the unstimulated background of 30 spots per million PBMCs.

### Systematic epitope mapping using pooled and individual FVIII peptides

**Figure 2:**
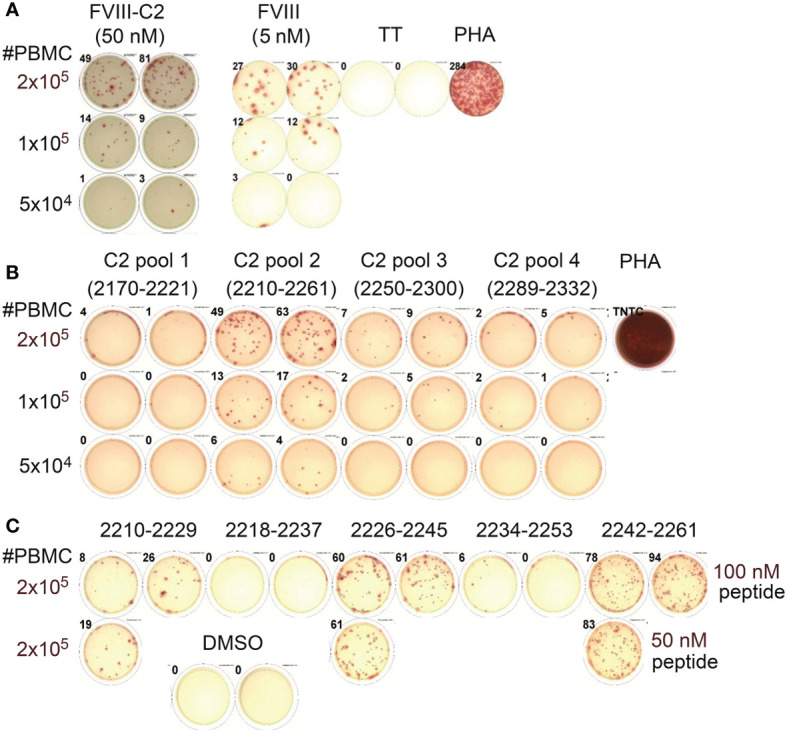
Mapping of CD4^+^ T-cell epitopes in the FVIII C2 domain by peptide ELISPOT assays (Int22Inv subject P1). **(A)** This subject’s PBMCs showed robust IFN-γ secretion when stimulated with rFVIII and rFVIII-C2 proteins. The FVIII-C2 ELISPOTs were carried out on different plates and read separately, hence the different background color in this image. **(B)** Decoding of his response to the FVIII C2 domain using pools of 20-mer peptides spanning the C2 domain sequence indicated that C2 pool #2 contained one or more immunodominant epitopes. **(C)** Further decoding by stimulations with the individual 20-mer peptides comprising C2 pool 2 indicated that he had specific responses to three epitopes in the FVIII C2 domain. His PBMCs did not respond to pooled peptides spanning the FVIII A2 domain (not shown).

Subjects P1 and P11 (both with an intron-22 inversion) generously donated sufficient blood to carry out fairly comprehensive epitope mapping by ELISPOT assays. P1 had a past inhibitor that had resolved (peak titer 15.2 BU/mL approximately 10 years prior to donating this sample). IFN-γ ELISPOT assays showed robust responses to both rFVIII and rFVIII-C2 protein (**Figure 2A**). His responses to A2 and C2 domain peptides were tested next, in order to test the hypotheses that tolerance to A2 epitopes was conferred by translation from the inverted *F8* mRNA (exons 1-22, containing the A2 domain sequence) and/or from *F8B* mRNA (exons 23-26, containing the C2 domain sequence). No responses to pooled A2 domain peptides were revealed under these assay conditions (not shown). However, his PBMCs responded to one of the pools of 20-mer peptides spanning the C2 domain sequence (**Figure 2B**). Decoding of this response by testing the individual peptides comprising this pool identified robust responses to peptides corresponding to C2-2210-2229, C2-2226-2245 and C2-2242-2261 (**Figure 2C**).

**Figure 3:**
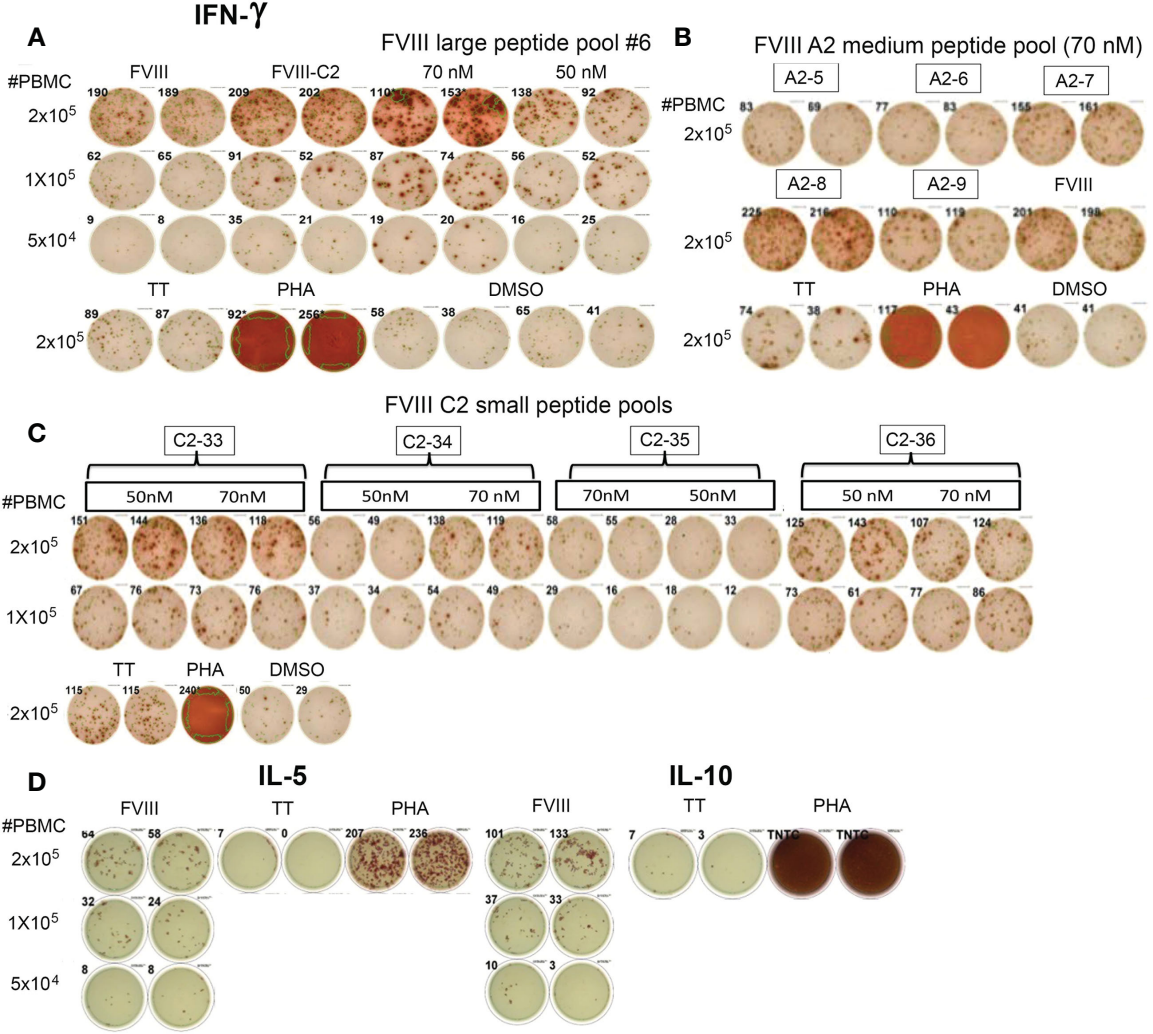
CD4^+^ T cells from Int22Inv subject P11 respond to epitopes in the FVIII A2 and C2 domains. **(A)** He showed a strong IFN-γ response to rFVIII, rFVIII-C2 protein, and FVIII large peptide pool #6 (which contained peptides spanning the C2 domain sequence). **(B)** His PBMCs also responded to FVIII A2 domain medium peptide pools A2-7, A2-8 and A2-9. **(C)** Decoding of his response to rFVIII-C2 protein and FVIII large peptide pool #6 by stimulating his PBMCs with small peptide pools spanning the FVIII C2 domain sequence. His PBMCs responded to epitopes in pools C2-33, C2-34 and C2-36. **(D)** This subject also showed Th2 (IL-5) and Tr1 (IL-10) responses to rFVIII stimulation. His responses to multiple epitopes in the A2 and C2 domains indicate that the inverted *F8* mRNA containing exons 1-22 and *F8B* mRNA (encoding the putative FVIIIB protein containing the FVIII C2 domain sequence) did not confer tolerance to the FVIII A2 or C2 domains. TT = tetanus toxoid. PHA = phytohemagglutinin. Negative control: DMSO (5 μl/well). rFVIII: 5 nM, rFVIII-C2: 50 nM.

Subject P11 also had a previous inhibitor that resolved four years before donating this blood sample. His PBMCs showed robust IFN-γ secretion when stimulated with rFVIII, rFVIII-C2 protein, peptide pools spanning the FVIII A2 domain sequence, and peptide pools spanning the FVIII C2 domain sequence (**Figures 3A–C**). His PBMCs also responded to rFVIII stimulation with secretion of IL-5 and IL-10 (**Figure 3D**).

### Querying Int22Inv T-cell responses to a known HLA-DRB1*1101-restricted epitope in FVIII

IFN-γ ELISPOT assays were carried out to determine if PBMCs from six severe HA subjects with an *HLA-DR11* allele would respond to a known HLA-DRB1*1101-restricted T-cell epitope in the FVIII A2 domain, FVIII-589-608, which was identified in a HA subject with missense substitution R593C (20). The overlapping 20-mer peptides FVIII-A2-28 and/or A2-29 both contained the epitope of interest. Two of the subjects had an Int22Inv mutation, two had a frameshift mutation, and the HA-causing mutation was unknown for the other two. One of the Int22Inv subjects responded to peptide A2-29, and both of the Int22Inv subjects also showed a robust response to the FVIII-C2 protein (**Figure S1**). Together, these results indicated the Int22Inv mutation did not confer tolerance to either the FVIII A2 or C2 domains.

### Mapping epitopes restricted to *HLA-DR3*

**Figure 4:**
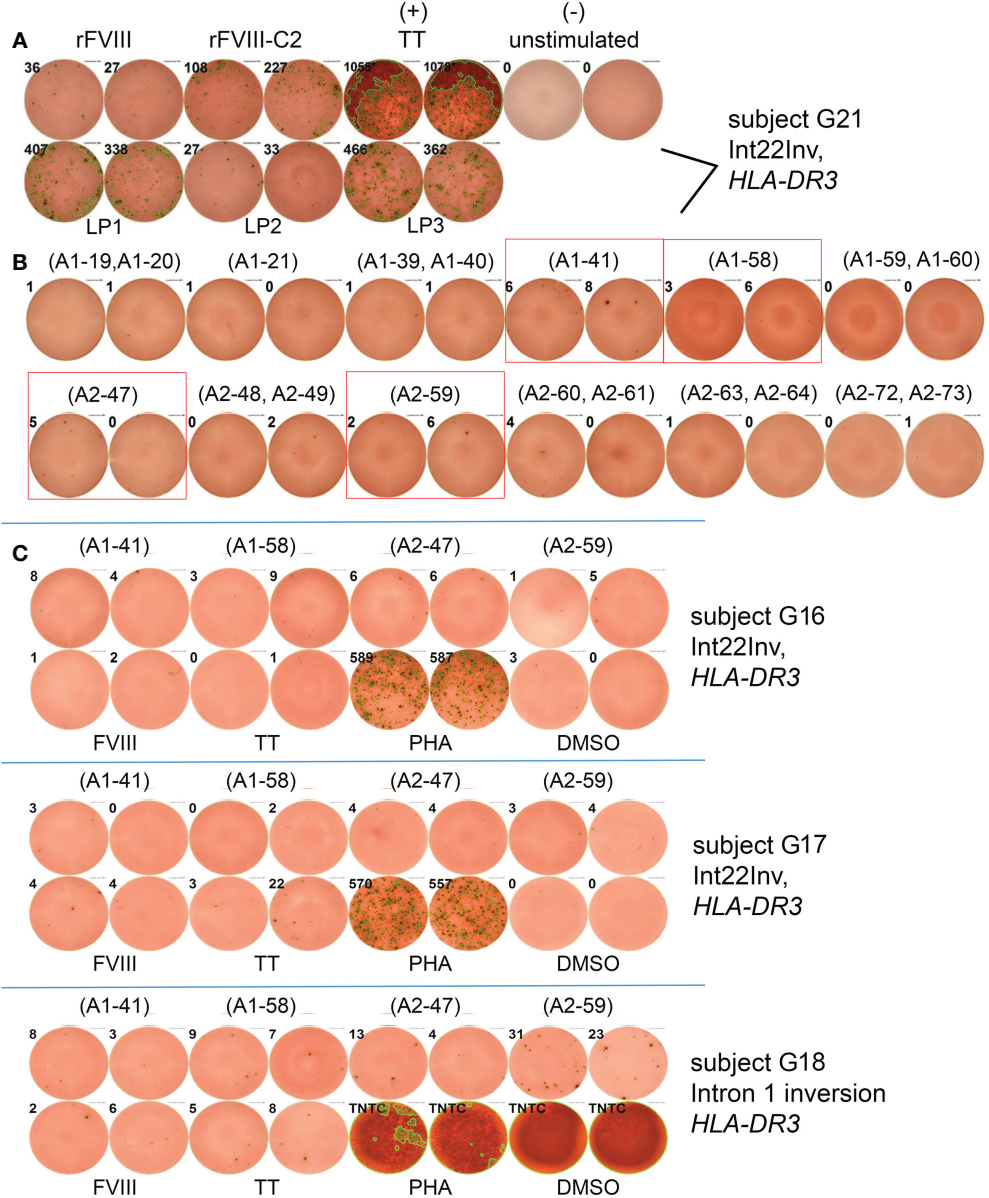
Epitope mapping of HLA-DRB1*0301-restricted responses to FVIII A1 and A2 domains by ELISPOT assays. **(A)** PBMCs from Int22Inv subject G21 responded to rFVIII and rFVIII-C2 domain protein, tetanus/diptheria toxin, and FVIII large peptide pools LP1, LP2 and LP3. Peptide pools LP1-LP3 consist of overlapping 15-mer peptides spanning the FVIII A1 and the A2 domains (**Table S3**). and LP2 **(B)** PBMCs from the same subject also responded to individual 15-mer FVIII peptides A1-41, A1-58, A2-47 and A2-59. Results for the remaining 6 peptides or peptide pools were negative and are not pictured. **(C)** PBMCs from an additional three Int22Inv subjects with an *HLA-DR3* allele (G16, G17 and G18) showed responses above background for one or more of the 4 FVIII A1 or A2 domain peptides. The response of the final subject to peptide FVIII-A2-59 (FVIII 605-619) was the most promising.

ELISPOT assays were first carried out for subject G21, who had an Int22Inv mutation and was monogenic for HLA-DRB1*0301. His PBMCs showed positive IFN-γ responses to rFVIII and to large peptide pools LP1, LP2 and LP3, which together span FVIII residues 1-737 (**Figure 4A**). The FVIII heavy chain consists of the A1 and A2 domains, residues 1-740. There were not enough PBMCs to allow systematic decoding *via* ELISPOT assays using smaller peptide pools and then individual peptides covering 740 residues. Therefore, the Immune Epitope Database (IEDB) server (33) was used to predict peptides within the FVIII A1 and A2 domains that would bind with high affinity to HLA-DR0301. Twenty-five 15-mer FVIII peptides from our library contained a total of 18 unique 9-mer motifs that were predicted to be in the top 2% for binding to HLA-DR0301 (**Table S8**). In several cases where overlapping 15-mer peptides contained the same motif the 2-3 peptides were pooled, generating a total of 18 individual or pooled FVIII peptides for further testing (**Table S9**). Stimulation of his PBMCs with four of these individual peptides, A1-41, A1-58, A2-47 and A2-59, produced IFN-γ secretion slightly above background levels (**Figure 4B**).

Next, PBMCs from three additional Int22Inv HA subjects who all had a current inhibitor and an *HLA-DR3* allele, G16, G17 and G18, were tested for IFN-γ responses to these 4 peptides by ELISPOT assays (**Figure 4C**). The strongest response was from G18, responding to peptide A2-59. Therefore, HLA-DR0301 tetramers loaded with the 4 pooled peptides, and with individual peptide A2-59, were ordered.

### Other HA ELISPOT assays

PBMCs from 11 severe and moderate HA subjects, three of whom had a confirmed Int22Inv mutation, were tested for responses to rFVIII and rFVIII-C2 protein (**Figure S2**). Eight of them showed robust cytokine secretion in response to rFVIII and 9 responded to rFVIII-C2. In general, comparing results of ELISPOT experiments testing reactivity to rFVIII and rFVIII-C2, among the positive responders IFN-γ secretion was usually more robust when cells were stimulated with 50 nM rFVIII-C2 than with 8 nM rFVIII. Unfortunately, FVIII protein becomes toxic to CD4 T cells at higher concentrations. The stronger responses to rFVIII-C2, and to pooled or individual FVIII peptides in some assays, may simply be due to stimulation of lower-avidity CD4 T cells by the higher concentrations of rFVIII-C2 and FVIII peptides. Of note, the rFVIII-C2 preps were routinely tested to confirm low endotoxin levels.

### Non-HA control ELISPOT assays

Control ELISPOT experiments indicated that IFN-γ secretion from non-HA PBMCs in response rFVIII stimulation under these assay conditions (**Figure S3**) was rare, while responses of non-HA PBMCs to rFVIII-C2 protein (included as a control in several experiments) were seen more frequently but still were not common.

### Background proliferation

Two severe HA and two non-HA control subjects showed high background IFN-γ secretion (seen in ELISPOT wells with DMSO alone added); they were therefore excluded from this study and are not included in any figures or tables.

### Epitope mapping by HLA Class II tetramer staining and isolation of FVIII-specific T-cell clones

HLA-Class II tetramer staining was carried out using CD4^+^ T cells isolated from four subjects with an Int22Inv mutation. Subject G16 was an adult inhibitor subject who had an HLA-DRB1*0301, 1201g haplotype. Subject G19 was an adult inhibitor subject with HLA-DRB1*0302, 0901 haplotype. Subject G20 (age range 4-17 years) had the HLA-DRB1*0301, 1101 haplotype and a current inhibitor. Subject G21 (age range 4-17 years) had the HLA-DRB1*0301, 0301 haplotype and no inhibitor history. Tetramer staining was also carried out for subject G18, an adult with an intron 1 inversion mutation, a current inhibitor, and HLA-DRB1*0301, 1401g haplotype, in order to further test for HLA-DRB1*0301-restricted epitopes in FVIII. Tetramer staining of CD4^+^ T cells isolated from these subjects’ PBMCs was followed by sorting of tetramer-hi cells and expansion of clones in culture.

### Tetramer staining to test for T-cell responses to epitopes in the FVIII A2 domain

**Figure 5:**
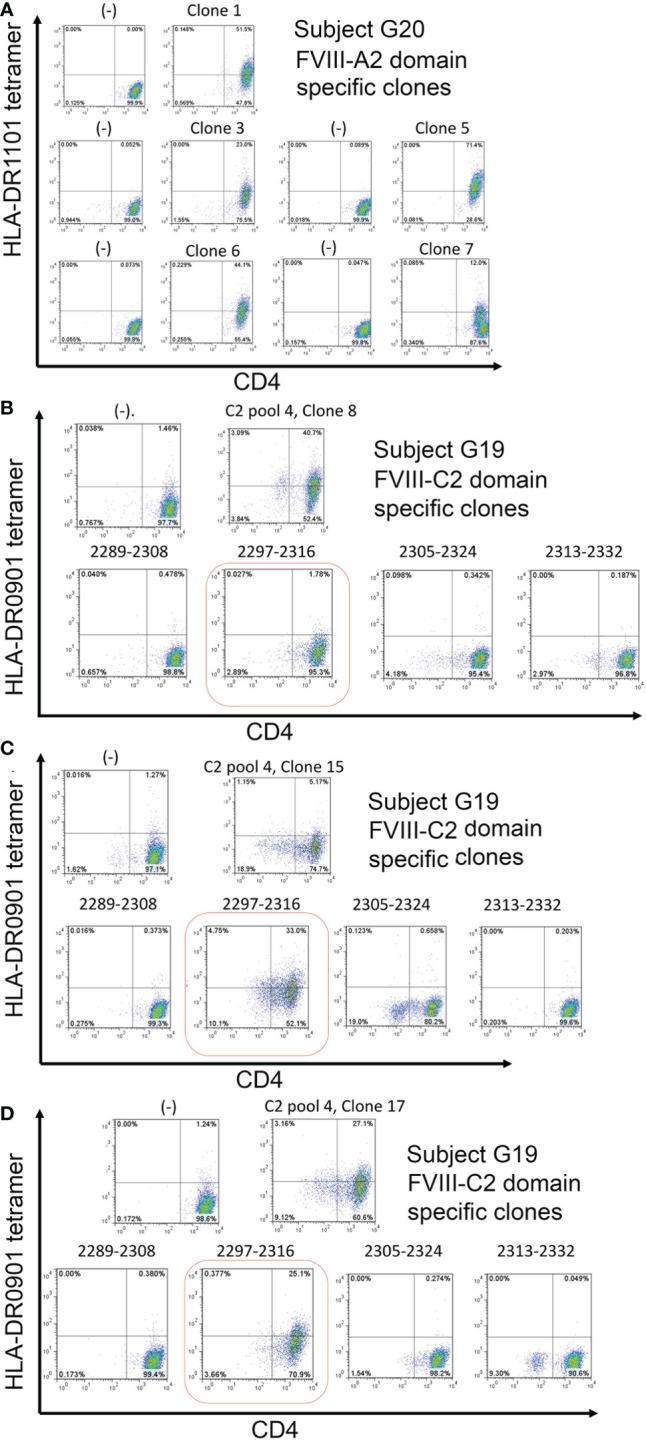
Tetramer staining identifies CD4^+^ T-cell clones restricted to FVIII A2 and C2 domain peptides. **A.** CD4^+^ T-cell clones restricted to one or more epitopes in the FVIII A2 domain were isolated from inhibitor subject G20 (Int22Inv, HLA-DRB1*0301, 1101) PBMCs by staining his CD4^+^ T cells with DR1101 tetramers loaded with A2 peptide pool #6 (four 20-mer peptides spanning FVIII residues 565-616). The tetramer-hi cells were then single-cell sorted, expanded in culture, and the expanded clones were stained with the same tetramer, following standard protocols in our laboratory (29). All five clones showed high-avidity tetramer binding**. B, C, D.** CD4^+^ T-cell clones recognizing pooled and individual peptides corresponding to the FVIII C2 domain were isolated from inhibitor subject G19 (Int22Inv, HLA-DRB1*0302, 0901) following a similar protocol. Clones recognizing C2 pool 4 peptides (spanning FVIII residues 2238-2332) were isolated. Decoding of this response using DR0901 peptides loaded with the individual peptides comprising this pool identified FVIII 2297-2316 as the immunodominant epitope. Negative controls: staining using the same HLA-DR tetramers loaded with irrelevant (tetanus) peptides did not produce tetramer-hi signals, indicating that these tetramers did not bind nonspecifically to CD4^+^ T cells.

PBMCs from Int22Inv subject G20 were queried using HLA-DR1101 tetramers loaded with peptide pool A2-6, which consisted of four 20-mer peptides spanning FVIII residues 565-616. This region contains a known HLA-DRB1*1101-restricted T-cell epitope that was recognized by HA subjects with an R593C missense mutation (20). **Figure 5A** shows staining of representative T-cell clones with HLA-DR1101 tetramers loaded with peptide pool A2-6, obtained following expansion of CD4^+^ T cells using this same peptide pool. Results clearly indicate recognition of one or more epitopes in the region FVIII 565-616.

### Tetramer staining to test for T-cell responses to epitopes in the FVIII C2 domain

CD4^+^ T-cell clones were isolated from Int22Inv subject G19 following expansion of CD4^+^ T cells using a 20-mer peptide pool spanning the FVIII C2 domain region 2289-2332. **Figures 5B–D** shows tetramer staining of representative HLA-DRB1*0901-restricted T-cell clones recognizing these pooled peptides. A second staining of expanded clones using tetramers loaded with the individual FVIII-C2 peptides comprising this pool identified FVIII-2297-2316 as an HLA-DRB1*0901-restricted T-cell epitope recognized by CD4^+^ T cells from this subject.

### *HLA-DR3*-restricted T-cell clones recognizing another FVIII A2 domain epitope

**Figure 6:**
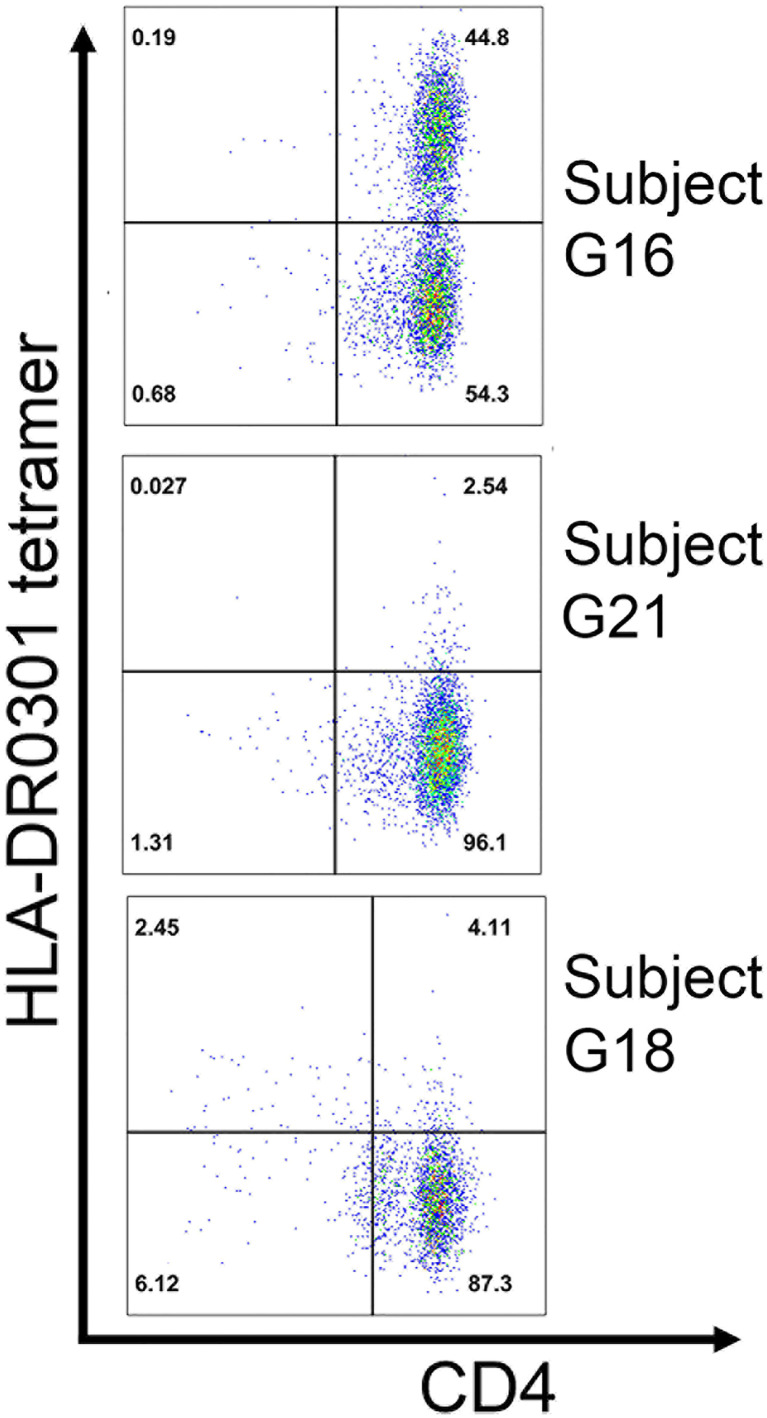
Confirmation of an HLA-DRB1*0301-restricted FVIII-A2 domain epitope by tetramer staining. T-cell clones expanded by stimulation with 15-mer peptide FVIII-A2-59 (FVIII 605-619) from three unrelated HA subjects: G16 (Int22Inv, current inhibitor), G18 (intron 1 inversion, current inhibitor), and G21 (Int22Inv, no inhibitor history). All three had an HLA-DRB1*0301 allele, and their clones were stained with an HLA-DR0301 tetramer loaded with peptide FVIII-A2-59. The results indicate that this peptide contains an immunodominant, HLA-DRB1*0301-resticted T-cell epitope recognized by CD4^+^ T cells from these subjects.

PBMCs from three Int22Inv subjects and one intron 1 subject were stimulated with a pool of four immunogenic peptides identified by ELISPOT assays: A1-41, A1-58, A2-47 and A2-59 (**Figure 4B**). The expanded cells were then stained and single-cell sorted using HLA-DR0301 tetramers loaded with these pooled peptides (not shown). Clones were obtained from three of these subjects. Specificity of the staining was then confirmed using an HLA-DR0301 tetramer loaded with peptide A2-59. This tetramer produced strong staining for two clones expanded from Int22Inv subject G16, two clones from Int22Inv subject G21, and three clones from intron 1 inversion subject G18, confirming that this peptide contained a FVIII epitope. Representative tetramer staining results are in **Figure 6**. The gating strategy and staining of the remaining clones are in **Figure S4**.

### Positive and negative controls for tetramer-based assays

Positive controls stimulating PBMCs from subjects with tetanus-diptheria toxoid peptides demonstrated the validity of tetramer-based epitope mapping *via* isolation of multiple TT-specific T-cell clones (**Figure S5**). Negative controls for all tetramer experiments consisted of tetramers loaded with an irrelevant peptide to rule out nonspecific binding of the tetramer to CD4^+^ T cells.

### Summary of FVIII immunogenicity assays

Results of all assays, grouped by assay type and presented as per-subject results, as well as clinical and demographic data, are summarized in **Table 4**.

**Table 4:** Per-subject summary of clinical, demographic and experimental data.

Subject code in database	HA severity	HA mutation	Age range	HLA-DRB1	Current inhibitor?	Past inhibitor that resolved?	Inhibitor peak (BU/mL)*	FVIII protein response?	FVIII-C2 protein or peptide response?	FVIII-A1 and/or A2 peptide response?	Exon 22-23 junction peptide response?	Clones?
Exon 22-23 junction peptide ELISPOTS (**Table 3**)												
G1	severe	Int22Inv + frameshift	18+	0701, 0701	N	N	0				N	
G2	severe	Int22Inv	4-17	0901, 1101g	N	N	0				N	
G3	severe	Int22Inv	18+	0701, 1301	Y	N	49	N			N	
G4	severe	Int22Inv	18+	0302, 1501	Y	N	0.6	Y			Y	
G5	severe	Int22Inv	4-17	0701, 1501	N	N	0	N			N	
G6	severe	Int22Inv	18+	0801, 1503	Y	N	68	N			N	
G7	severe	Int22Inv	4-17	0404, 1301	N	N	0				N	
G8	severe	Int22Inv	4-17	0401, 0701	Y	N	1	N			N	
G9	severe	Int22Inv	18+	0102, 1503	Y	N	>1000	N	Y		N	
												
HLA-DR11, DR1 or DR15 alleles - A2-589-608 peptide ELISPOTS (**Figure S1**)												
G10	severe	Int22Inv	18+	1101, 1503	N	N	0	N	Y	Y		
G11	severe	unknown	4-17	1101, 1602	Y	N	47	N	Y	N		
G12	severe	Int22Inv	18+	1101, 1101	N	N	0	N	Y	N		
G13	severe	unknown	18+	1101, 1301	Y	N	32	N	N	N		
G14	severe	frameshift	4-17	0701, 1104	N	N	0	N		N		
G15	severe	frameshift	4-17	0301, 1104	Y	Y	unknown	N		N		
HLA-DR3 - A1 and A2 domain peptide ELISPOTS (**Figure 4**)												
G16	severe	Int22Inv	18+	0301, 1201g	Y	N	31	N		N		
G17	severe	Int22Inv	18+	0301, 0804	Y	N	17	N		N		
G18	severe	Intron 1 inversion	18+	0301, 1401g	Y	N	80	N		Y		
G21	severe	Int22Inv	4-17	0301, 0301	N	N	0	Y		Y		
FVIII and FVIII-C2 protein ELISPOTS (**Figure S2**)												
P1	moderate	Int22Inv	18+	unknown	N	Y	15.2	Y	Y			
P2	moderate or severe	unknown	18+	unknown	unknown	unknown	unknown	Y	Y			
P3	severe	frameshift deletion	18+	unknown	Y	N	89.6	Y	Y			
P4	moderate or severe	unknown	18+	unknown	unknown	unknown	un known	Y	N			
P5	severe	Int22Inv (atypical)	18+	unknown	Y	N	716.8		Y			
P6	moderate or severe	unknown	18+	unknown	unknown	unknown	unknown		Y			
P7	severe	base substitution	18+	unknown	N	Y	51.2	Y	Y			
P8	moderate	unknown	18+	unknown	Y	N	384	N	N			
P9	moderate	unknown	18+	unknown	Y	N	4.6	Y	Y			
P10	severe	unknown	18+	unknown	N	Y	6	Y	Y			
P11	severe	Int22Inv	18+	unknown	N	Y	unknown	Y	Y			
Tetramer isolation of CD4 T-cell clones (**Figures 5**, **6**, **S4**, **S5**)												
G19	severe	Int22Inv	18+	0302, 0901	Y	N	3456					C2-specific
G20	severe	Int22Inv	4-17	0301, 1101	Y	N	0					A2-specific
G21	severe	Int22Inv	4-17	0301, 0301	N	N	0					A2-specific
G16	severe	Int22Inv	18+	0301, 1201g	Y	N	31					A2-specific
G18	severe	Intron 1 inversion	18+	0301, 1401g	Y	N	80					A2-specific
Normal control subjects (**Figure S3**)												
NC1	n/a	n/a	18+	unknown	n/a	n/a	n/a	N				
NC2	n/a	n/a	18+	unknown	n/a	n/a	n/a	N				
NC3	n/a	n/a	18+	unknown	n/a	n/a	n/a	N				
NC4	n/a	n/a	18+	unknown	n/a	n/a	n/a	N				
NC5	n/a	n/a	18+	unknown	n/a	n/a	n/a	N				
NC6	n/a	n/a	18+	unknown	n/a	n/a	n/a	N				

*Bethesda units (BU)/mL.

## Discussion

The hypothesis that HA patients with an intron-22 inversion (Int22Inv) mutation have a lower risk of developing a neutralizing anti-FVIII antibody (“inhibitor”) response (24, 25) has gained fairly wide traction in the hemophilia A community. Well-conducted earlier studies have indeed indicated that severe HA patients with an intron-22 inversion mutation had a lower inhibitor incidence compared to patients with other large structural changes in the *F8* gene such as large frameshifts or deletions, early stop codons, etc. (22, 23). However, an important point to note is that the sizes of these respective HA cohorts differ substantially: almost half of severe HA patients have an intron-22 inversion mutation, while the other large structural changes are a heterogeneous group of mutations that together comprise only ~6% of all mutations resulting in severe HA (34) (Ahmed and Pratt, J Thromb Haemost, *in press*). Furthermore, a significant fraction of patients with specific, rare large structural *F8* changes are related, compared to the overall low relatedness among the intron-22 inversion population. A family history of inhibitor development has been noted as a risk factor in multiple studies, indicating roles for other genetic factors influencing immune and/or inflammatory responses (35–37).

Individuals with an Int22Inv mutation express an mRNA from the inverted locus that contains *F8* exons 1-22 spliced to an additional 16 in-frame codons, followed by a stop codon (1, 2). An alternative *F8* isoform expressed in multiple human tissues, termed *F8B* mRNA, is a 2.6-kb transcript initiated from a start site within intron 22 and containing *F8* exons 23-26 (4). HA-Int22Inv patients do not circulate measurable FVIII antigenic material (often referred to as cross-reactive material, or FVIII-CRM+). Almost all of them have FVIII clotting activity (FVIII:C) levels <1% normal, i.e., by definition they have severe HA. (The rare exceptions, which generally report FVIII levels of 1-2% normal, are likely due to experimental variations or unusually high activity of other non-FVIII clotting factors). It has been hypothesized that partial FVIII proteins are expressed intracellularly from these two partial *F8* transcripts, and that they contain FVIII residues 1-2124 (encoded by *F8* exons 1-22) and FVIII residues 2125-2332 (encoded by *F8B* mRNA containing *F8* exons 23-26) (24, 38). It has been further hypothesized that this intracellular expression of the entire FVIII sequence, contained in 2 partial FVIII proteins, confers immune tolerance to these proteins, thereby explaining the apparently lower inhibitor risk associated with Int22Inv mutations (25). If such tolerance is conferred to Int22Inv patients, then their CD4^+^ T-cell responses (providing help for anti-FVIII antibody production) to infused, therapeutic wild-type FVIII would be restricted to a neoepitope containing FVIII residues M2124 and V2125, as the mRNA encoding this short region is interrupted by the inversion mutation, precluding translation of this region (24, 25). In support of this hypothesis, Pandey et al. reported detection of intracellular FVIII-CRM^+^ in both liver tissues and circulating cells from Int22Inv and nonhemophilic subjects, using antibody staining and LC-MS/MS analysis of cellular immunoprecipitates (24, 38). Our laboratory carried out similar experiments, using carefully validated antibodies to evaluate human and canine Int22-Inv liver tissues and cellular samples *via* immunofluorescence, immunohistochemistry, western blots and LC-MS/MS of immunprecipitates. We have been unable to detect intracellular expression of FVIII-CRM^+^ proteins using these sensitive assays, leading us to suggest that antibodies used in the earlier studies were in fact binding nonspecifically to other antigens besides FVIII (39).

The most relevant data addressing the question of possible tolerance to FVIII is the actual patient outcomes. Our lab recently carried out a systematic regression analyses of data from >6,000 HA subjects enrolled in the “My Life Our Future” study in the U.S., of whom 1,075 had an Int22Inv mutation (Ahmed and Pratt, J Thromb Haemost, *in press*). A major conclusion of this study was that inhibitor risk associated with Int22Inv mutations was indistinguishable from that associated with other large structural changes in the *F8* gene. We attribute the apparent discrepancy of this result with reports from earlier case-control and meta-analysis studies (22, 23) to heterogeneity in the respective, much smaller cohorts (compared to the Int22Inv cohort) in each study that had mutations entailing large structural *F8* changes. Thus, the recently obtained statistical data from a large cohort in the U.S. indicate that individuals with an Int22Inv mutation are as likely to develop an inhibitor response as those with HA due to other major *F8* gene disruptions.

The present study directly addresses the question of whether Int22Inv patients are tolerized to FVIII, with the exception of a hypothesized neoepitope encoded by the *F8* exon 22-23 junction region. First, the binding affinities of 20-mer peptides spanning the exon 22-23 junction regions to ten recombinant HLA-DR proteins were determined using an established peptide-HLA competition binding assay (17, 29, 31). Predicted peptide-HLA-DR affinities were also obtained using a recent update of the same algorithm (MHCIIPan) used by Sauna et al. to predict the immunogenicity of this region (30). As in their study, medium-to-strong affinity binding of these peptides to multiple HLA-DR was predicted. However, the peptide binding assays revealed far fewer high- or medium-affinity interactions (**Figure 2**). Our experimental results showed high- or medium-affinity MHCII binding of these peptides by HLA-DR7, DR9 and DR15, but not by the other 7 HLA alleles that were tested; these 10 HLA-DR alleles were broadly representative of the U.S. population.

Although the IEDB and other prediction algorithms are extremely useful for applications such as determining prior exposure to a given pathogen or antigen, or peptide-based vaccine design, or for generating candidates for experimental tests of peptide immunogenicity, experimental validation is important before investing too many resources on the basis of predictions alone. MHC Class II and T-cell epitope prediction algorithms are continually being improved, and data such as those in the present study, characterizing both binding and non-binding of peptides to specific MHC alleles, can be utilized to train prediction algorithms and further improve their accuracy (40). Based on the present study’s experimental results, we conclude that the exon 22-23 junction-encoded region, encompassing the FVIII C1 domain sequence extending 9-12 residues on either side of M2124-V2125, is unlikely to comprise an immunodominant, promiscuous T-cell epitope driving anti-FVIII immune responses in most HA-Int22Inv patients. The one subject who showed positive ELISPOT results (IFN-γ secretion in response to stimulation with exon 22-23 junction peptides), subject G4, had a current low-titer inhibitor, and this result indicated that an epitope in the exon 22-23 junction region contributed to his anti-FVIII immune response. He also had a missense *F8* mutation, H1499Y, although the relevance of this second mutation in an individual with an Int22Inv mutation is not clear, given that FVIII intact would not be expressed. The mutation H1499Y was not found in the CHAMPS hemophilia A mutation database (34) (accessed 01/24/2023, https://www.cdc.gov/ncbddd/hemophilia/champs.html), so we found no independent data regarding a potential association with inhibitor development. The remaining eight Int22Inv subjects (one also had a frameshift mutation) did not secrete IFN-γ in response to stimulation with the exon 22-23 junction peptides (**Tables 3**, **4**).

Do individuals with an Int22Inv mutation have immune tolerance to FVIII proteins encoded by inverted *F8* exons 1-22 and/or *F8B* exons 23-36? This question was addressed by experiments to test the null hypothesis using independent, complementary methods: ELISPOT assays and HLA-DR tetramer staining. CD4^+^ T-cell responses to rFVIII proteins and peptides were queried using PBMCs obtained from HA-Int22Inv subjects, as well as HA subjects with other *F8* mutations and healthy non-HA controls. Positive controls for these assays included stimulation with tetanus/diptheria toxin (TT) and phytohaemagluttinin (PHA), while negative controls included stimulation with the dimethylsulfoxide (DMSO) carrier solution for peptides, or incubating cells with an irrelevant peptide-loaded tetramer, or comparisons of Int22Inv cellular responses with those of healthy non-HA normal controls. The ELISPOT assays showed interferon-γ secretion in response to rFVIII and/or rFVIII-C2 proteins in almost all of the HA-Int22Inv experiments, whereas anti-FVIII responses were rare in the non-HA control samples. Further epitope mapping using pooled and individual FVIII peptides as stimulants clearly identified HA-Int22Inv immune responses to the FVIII A2 and C2 domains.

HLA Class II tetramer staining was carried out as a stringent, independent test to identify HLA-restricted CD4^+^ T-cell responses to specific epitopes in FVIII, using PBMCS from Int22Inv subjects. An HLA-DRB1*1101-restricted epitope in FVIII was characterized earlier, identifying the wild-type FVIII A2 domain sequence 498-503 as a neoepitope recognized by CD4^+^ T cells from two unrelated HA subjects with missense mutation FVIII-R593C (20). Thus, using tetramers loaded with FVIII-A2 peptides, we were able to test the hypothesis that a subject with an Int22Inv mutation and HLA-DRB1*1101 allele would be tolerized to a confirmed HLA-DRB1*1101-restricted epitope that contributed to the anti-FVIII immune responses of HA subjects with a missense mutation at this site. Staining using HLA-DR1101 tetramers loaded with pooled A2 domain peptides, followed by isolation of multiple CD4^+^ T-cell clones, indeed confirmed that Int22Inv subject G21 responded to one or more HLA-DRB1*1101-restricted epitopes within the FVIII A2 domain region 565-616 (**Figure 5A**).

Staining of CD4^+^ T cells from a second Int22Inv subject, G19, using HLA-DR0901 tetramers loaded with pooled C2 domain peptides, produced positive staining using C2 peptide pool #4 (containing peptides spanning FVIII 2289-2332). Multiple clones were again isolated. Three of these pooled peptide responses were decoded by a second staining using tetramers loaded with the individual FVIII-C2 peptides comprising the pool. All three decoding experiments identified FVIII-2297-2316 as an HLA-DRB1*0901-restricted T-cell epitope contributing to the anti-FVIII T-cell response of this subject (**Figures 5B–D**). Finally, FVIII-specific T-cell clones recognizing an HLA-DRB1*0301-restricted epitope in the FVIII A2 domain were isolated from two Int22Inv subjects and one intron 1 inversion subject with the same *HLA-DRB1* allele (**Figure 6**). One of the Int22Inv subjects had a current inhibitor, one had no inhibitor history, and the intron 1 inversion subject had a current inhibitor. Two of these subjects also responded to FVIII A2 domain epitopes in ELISPOT assays (**Figure 4**). To summarize, CD4^+^ T-cell clones recognizing epitopes in the FVIII A2 or C2 domain were isolated from four unrelated Int22Inv subjects.

Approximately 20 years ago, the Conti-Fine group characterized CD4^+^ T-cell responses to FVIII in HA and non HA subjects, primarily through T-cell proliferation and ELISPOT assays employing FVIII and pools of synthetic FVIII peptides spanning several FVIII domains (10, 12–15, 41). The present study builds on their earlier work, focusing on specific epitopes recognized by HA subjects with an Int22Inv mutation. We also tested the hypothesis that HA-Int22Inv patients have been tolerized to FVIII sequences encoded by mRNAs containing *F8* exons 1-22 and/or 23-26. Results of this study provide evidence in support of the null hypothesis: rather than being tolerized, CD4^+^ T-effector cells from multiple Int22Inv subjects readily responded to multiple epitopes in FVIII. Together with recent statisitical/epidemiological evidence (Ahmed and Pratt, J Thromb Haemostas, *in press*) and our failure to detect FVIII-CRM^+^ proteins in liver tissues or circulating cells from Int22Inv subjects (39), the present study indicates that Int22Inv patients should be monitored just as closely as other severe HA patients for development of an inhibitor, especially during initial FVIII infusions.

## Data availability statement

The original contributions presented in the study are included in the article/**Supplementary Material**. Further inquiries can be directed to the corresponding author.

## Ethics statement

The studies involving human participants were reviewed and approved by Uniformed Services University IRB #1. Written informed consent to participate in this study was provided by the participants or by their legal guardian/next of kin.

## Author contributions

KP: Conceived the project and designed experiments, analyzed data, supervised the project and wrote the paper. DG, PV and AK: Performed experiments, analyzed data and edited the paper. MR: Enrolled subjects, consulted, and edited the paper. All authors contributed to the article and approved the submitted version.
