# Supplementary material for: Hemophilia A subjects with an intron-22 gene inversion mutation show CD4+ T-effector responses to multiple epitopes in FVIII
Source: Front Immunol. 2023 Mar 1;14:1128641. doi: 10.3389/fimmu.2023.1128641 (PMC10015889; doi:10.3389/fimmu.2023.1128641)
Supplement: Supplementary file 1 [file DataSheet_1.pdf]

Supplemental Data

Figure S1

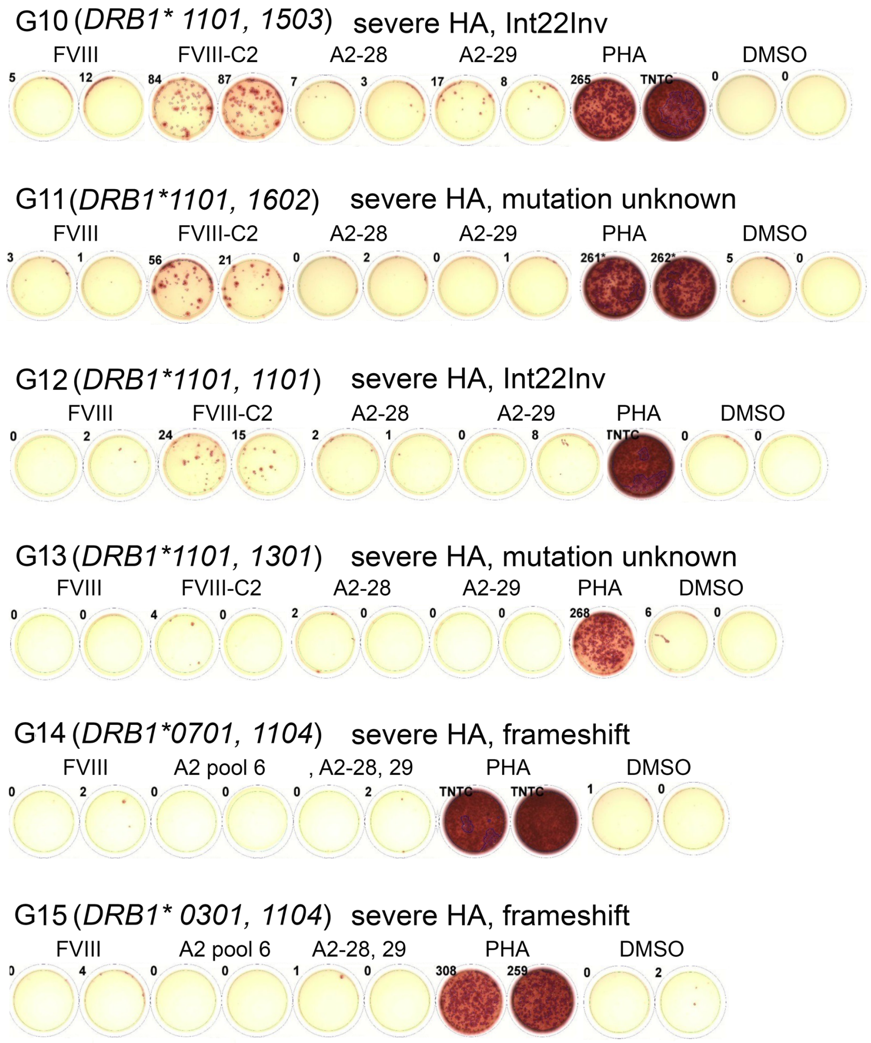

Figure S1. *IFN-γ ELISPOT assays to test for responses to a known HLA-DR11-restricted T-cell epitope in FVIII. All 6 subjects had an HLA-DR11 allele.* Each row shows wells from a single ELISPOT plate containing $2x10^5$ PBMCs. Peptide pool A2-6 contained 20-mer peptides spanning FVIII residues 565-616. Peptides A2-28 and A2-29 correspond to FVIII 581-600 and FVIII 589-608, respectively. These peptides both include the *HLA-DRB1*1101*-restricted epitope identified by James et al., FVIII 589-608, via tetramer staining of CD4 T cells and clones isolated from two unrelated HA subjects with a FVIII-R593C mutation (ref 20). This earlier study also showed, by peptide-HLA-DR binding assays, that the R593-608 motif bound with micromolar affinity to recombinant HLA-DR1101, DR0101 and DR1501 proteins. Note that PBMCs from the 2 known Int22Inv subjects tested for responses to FVIII-C2 protein showed robust IFN-γ secretion. Subject G10 also showed a positive response to peptide A2-29.

Figure S2

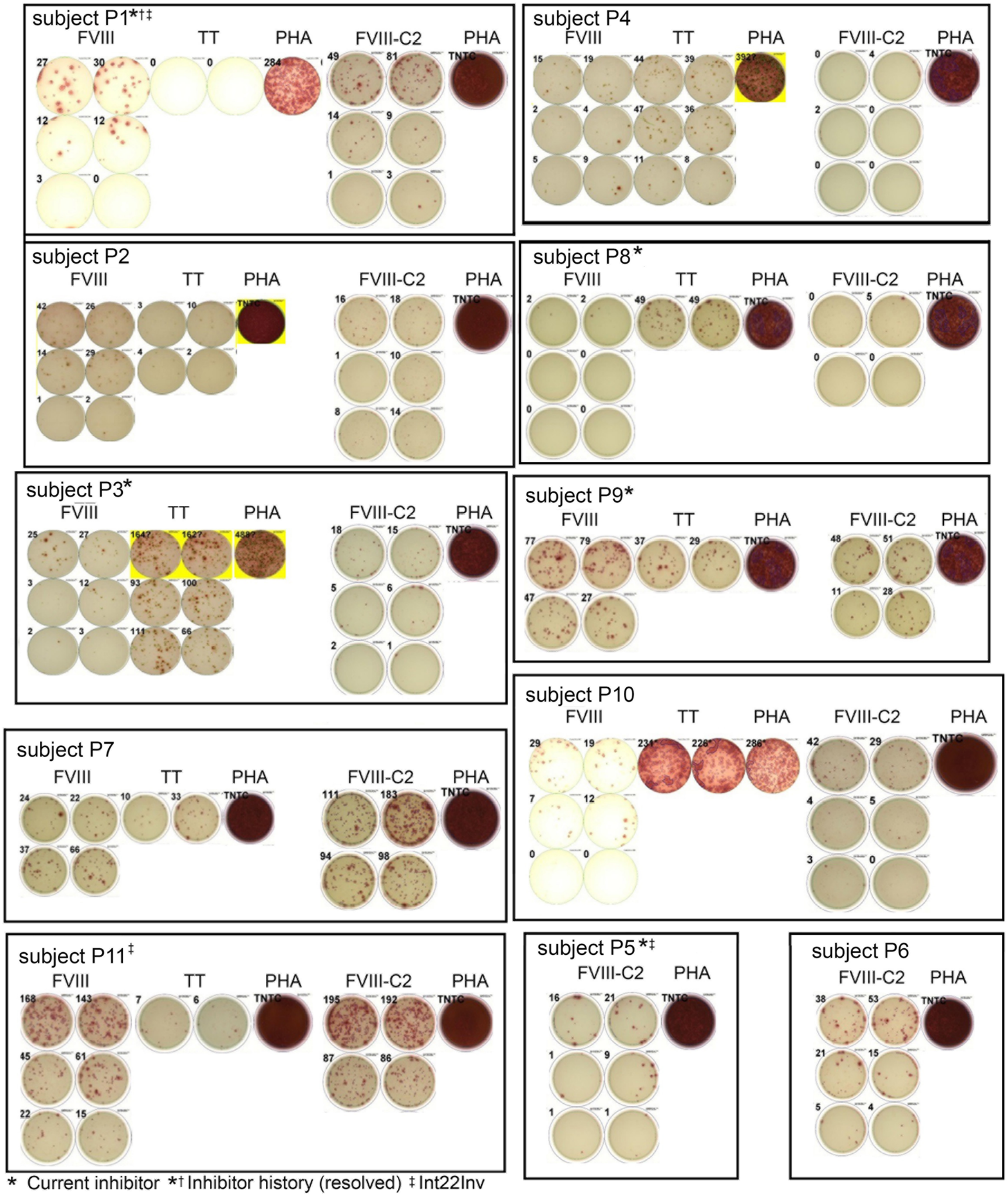

Figure S2. *PBMCs from nine of 11 severe and moderate HA subjects secreted IFN-γ in response to recombinant FVIII-C2 protein*. Subjects P4 and P8 did not show responses to FVIII-C2 protein. Representative results showing responses to stimulation with 5 nM FVIII and/or 50 nM FVIII-C2 protein. The stimulations were carried out in duplicate serial dilutions in wells containing $2x10^5$, $1x10^5$ and $5x10^4$ PBMCs, when sufficient cells were available

Figure S3

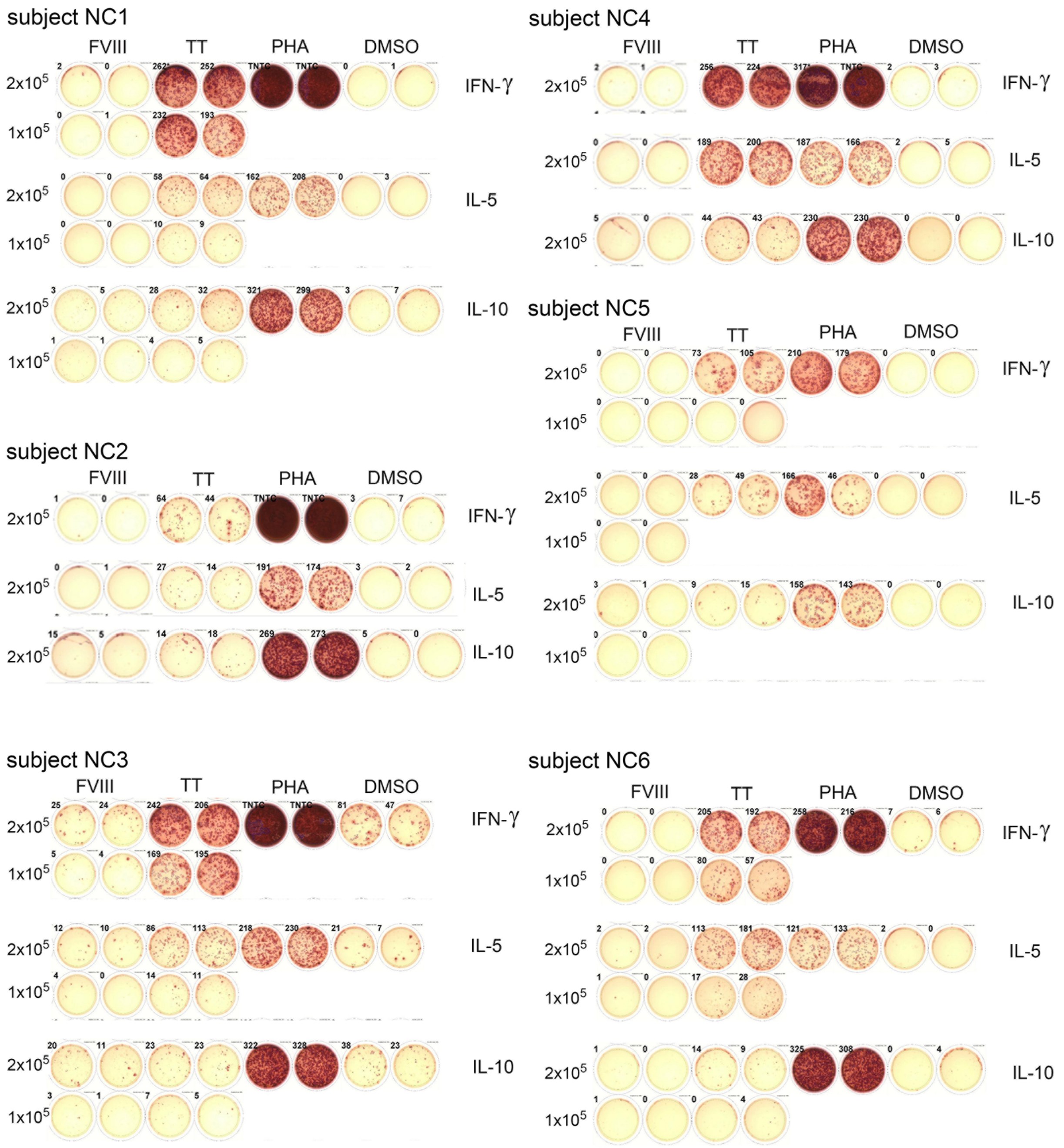

Figure S3. *Healthy non-HA subjects did not respond to FVIII stimulation under these assay conditions.* Representative results showing FVIII ELISPOT assays carried out using PBMCs from six healthy non-HA subjects. None of these subjects showed secretion of IFN-γ, IL-5 or IL-10 above background levels when their PBMCs were stimulated with 5 nM FVIII protein. Positive controls: Tetanus diptheria toxoid (TT) and phytohaemagluttinin (PHA). Negative control: 5 μL/well DMSO.

Figure S4

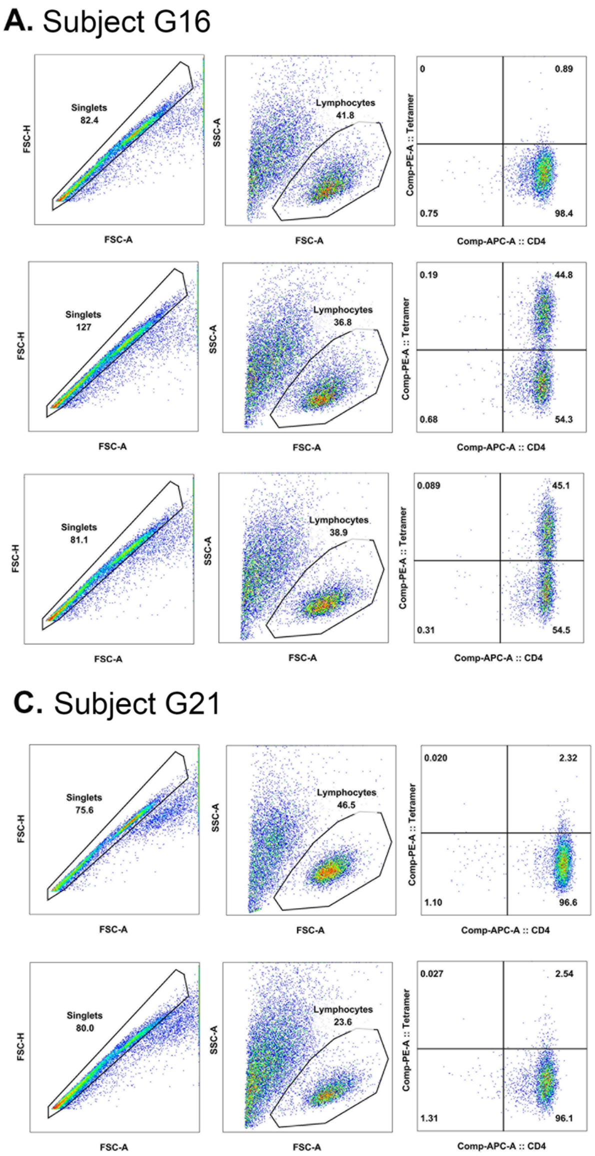

Figure S4. Gating strategy for isolation of *HLA-DRB1*0301*-restricted T-cell clones using tetramers loaded with 15-mer peptide FVIII-A2-59 (FVIII 605-619). Representative clones from each of these 3 subjects are shown in Figure 6.

Figure S5

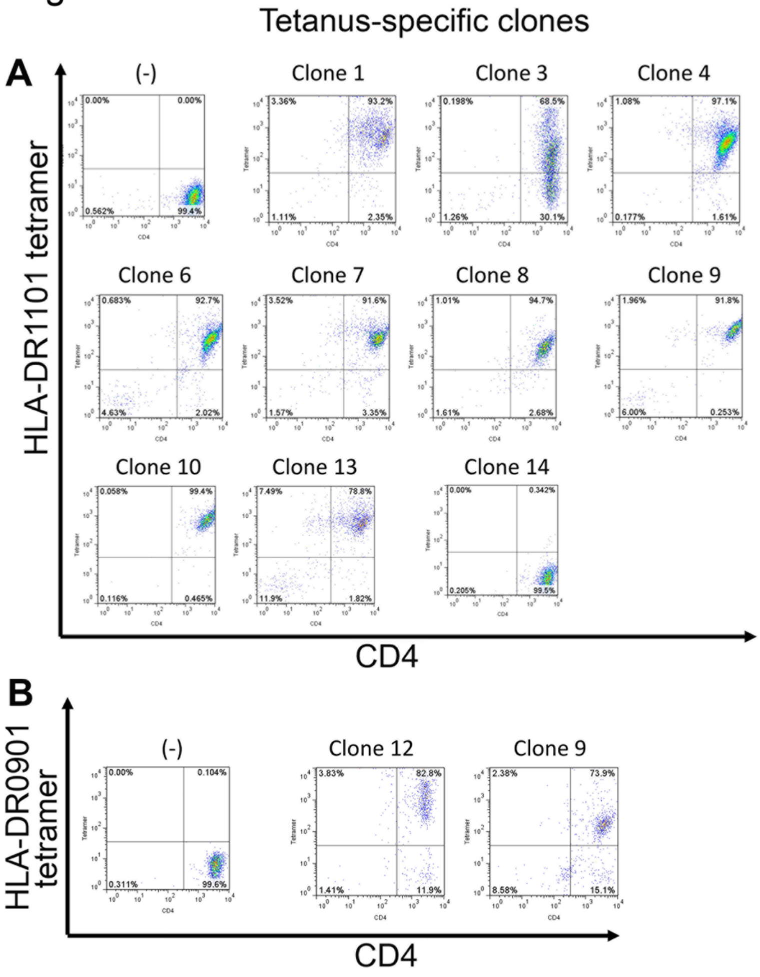

Figure S5. *Positive and negative controls for tetramer-based isolation and staining of clones.* **A.** CD4 T-cell clones restricted to tetanus peptide TT-946-965 were isolated from subject G21 (Int22Inv, *HLA-DRB1*0301, 1101*) PBMCs by staining his CD4 T cells with DR1101 tetramers loaded with this peptide, followed by single-cell sorting of tetramer-hi cells, expansion of clones in culture, and then staining the expanded clones with the same tetramer. Nine of the ten tetanus-specific clones showed high-avidity tetramer binding. Negative control: staining of one clone with a DR1101 tetramer loaded with an irrelevant peptide. **B.** Following the same protocol, CD4 T-cell clones restricted to tetanus peptide 498-517 were isolated from subject G19 (Int22Inv, *HLA-DRB1*0301, 0901*) PBMCs by staining his CD4 T cells with DR0901 tetramers loaded with this peptide, and isolation of clones as in (A).
